# Anorexia Nervosa in Juvenile Systemic Lupus Erythematosus (SLE): A Causality Dilemma

**DOI:** 10.3390/children10040697

**Published:** 2023-04-07

**Authors:** Maria G. Grammatikopoulou, Vasiliki Syrmou, Maria-Lydia Lioliopoulou, Konstantinos Gkiouras, Theodora Simopoulou, Christina G. Katsiari, Tonia Vassilakou, Dimitrios P. Bogdanos

**Affiliations:** 1 Unit of Immunonutrition and Clinical Nutrition, Department of Rheumatology and Clinical Immunology, Faculty of Medicine, School of Health Sciences, University of Thessaly, Biopolis, GR-41110 Larissa, Greece; 2 Department of Public Health Policy, School of Public Health, University of West Attica, 196 Alexandras Avenue, GR-11521 Athens, Greece

**Keywords:** neuropsychiatric lupus, depression, feeding and eating disorder, leptin, mental health, weight loss, body image, pediatric rheumatology, trauma, weight loss

## Abstract

Juvenile-onset systemic lupus erythematosus (jSLE) is an autoimmune disorder with multifaceted clinical findings in different organ systems. Neuropsychiatric manifestations affect more than half of SLE patients, and there is increasing evidence that anorexia nervosa (AN), a feeding and eating disorder (FED) characterized by significantly reduced energy intake, is among them. Herein, a review of the literature on the potential association between jSLE and AN was performed. Reported clinical cases were identified, and putative pathophysiological mechanisms were sought that could potentially explain the observed relationship between these two pathological entities. Four reports of isolated cases and a case series including seven patients were identified. In this limited patient pool, the diagnosis of AN preceded that of SLE in the majority of cases, whereas in all cases both entities were diagnosed within a time span of two years. Many explanations for the observed relationships have been proposed. AN has been associated with the stress of chronic disease diagnosis; on the other hand, the chronic inflammation associated with AN may contribute to the development/appearance of SLE. Adverse childhood experiences, concentrations of leptin, shared autoantibodies, and genetic traits appear to be important factors in this well-established interplay. In essence, it seems important to increase clinician awareness of the concomitant development of AN and SLE and invite further research on the subject.

## 1. Introduction

Systemic lupus erythematosus (SLE, lupus) is a polygenic autoimmune inflammatory rheumatic disease with great diversity in its clinical manifestations and the involvement of various tissues and organs of the body [1]. Apart from the diverse clinical symptoms, SLE is also associated with a plethora of autoimmune phenomena, which can even change during the course of the disease. Juvenile-onset SLE (jSLE) in particular, is presented before the age of 18 years and represents approximately 20% of the total SLE patient pool [2]. According to the Centers for Disease Control (CDC), the incidence of lupus has tripled during the past 40 years [3]. As with SLE, jSLE shows a predilection for women, thus being more prevalent among girls compared to boys [4].

SLE can cause significant disability, multiplying the morbidity and mortality risks of patients [5,6,7], with the juvenile-onset form being associated with even greater mortality risk compared to the adult form [8]. Common clinical manifestations involve fever, joint pain and arthritis, oral ulcerations, alopecia, malar rash, photosensitivity, and Raynaud’s phenomenon [9,10,11,12,13]. Organ-system involvement in SLE can include the musculoskeletal system and the skin, the kidneys, lungs, heart, and many more [9,14]. Among the distinct onset subtypes, jSLE is associated with a more severe phenotype, a more diverse clinical and serological profile, and extensive organ damage [15,16].

A variety of different pathogenic mechanisms converge toward the development of the distinct SLE clinical phenotype [1,10]. Although the exact etiology of lupus remains largely unraveled, the onset of jSLE is dependent on the synergy of several factors, including environmental triggers, genetic predisposition, stochastic factors, as well as immunological and hormonal specificities [17]. With specific environmental exposures, the underlying proinflammatory stimuli may cascade the loss of immune tolerance into self-antigens, driving autoimmunity [17,18].

### 1.1. Neuropsychiatric (NP) Lupus

In cohorts of patients with SLE, more than half eventually suffer from neuropsychiatric (NP) manifestations during the course of the disease [19,20]. The clinical features of these NP manifestations are also heterogenous and may include psychosis, headaches, cerebrovascular insults, seizures, depression, anxiety disorders, or cognitive dysfunction [19,20,21,22,23]. Due to this high prevalence of psychiatric conditions in SLE, the term NPSLE was coined, to encompass the mosaic of NP involvement in lupus, spanning from the central to the peripheral nervous system [24,25,26,27].

It is estimated that in 30% of patients exhibiting NP symptoms, SLE is the primary diagnosis, thus NP manifestations are usually presented around the time of SLE onset [26]. On the other hand, psychotic and neurovascular disorders may also lead to the development of NPSLE [26], indicating the existence of a bidirectional relationship between NP symptoms and SLE.

NP involvement is not limited to adult-onset SLE, with 22–95% of patients having a jSLE diagnosis exhibiting NP symptoms, including hallucinations, headaches, mood disorders, stroke and weakness, cognitive impairment, anxiety, seizures, movement disorders, peripheral neuritis, transverse myelitis, or cerebrovascular disease [15,28,29]. In fact, NP is more aggressive and severe in jSLE compared to in patients with adult-onset lupus, further multiplying the morbidity for young patients [8,30,31,32]. Disease activity indexes are markedly elevated in those with NPSLE, pointing to a direct relationship between inflammation and NP manifestations [28]. As a result, inflammation, ischemia, oxidative stress, microglial activation, and mitochondrial disorders, as well as dysfunction of the blood–brain barrier have been suggested as non-specific biomarkers for NPSLE [22]. Depending on the NP manifestation, NPSLE may increase the risk of mortality of patients [33], mainly as the result of cardiovascular disease (CVD) or organic brain syndrome.

### 1.2. Anorexia Nervosa (AN)

Anorexia nervosa (AN) is a feeding and eating disorder (FED) characterized by a significant restriction in the energy intake relative to one’s requirements, resulting in extreme low body weight for a person’s sex, age, developmental trajectory, and physical health [34]. This restriction in energy consumption is propelled by a distorted body image and an intense fear of gaining weight or being overweight/obese [34,35]. According to the 5th edition of the Diagnostic and Statistical Manual of Mental Disorders (DSM-V) [34], AN symptomatology is highly heterogenous, including changes in the anthropometry (low body weight), physiological (lanugo, tooth erosion, disrupted hormone regulation, etc.), as well as cognitive–behavioral symptoms (distorted body image, low self-esteem, etc.)

The peak onset occurs at mid adolescence [36], and recently a significant increase in the adolescent incidence of AN has occurred [37]. Among all mental diseases, AN is associated with the highest mortality [38] and the greatest disability rates [39]. Apart from the typical symptoms, the condition can lead to life-threatening situations, with severe hypokalemia and significant Q-T interval prolongation being some of the commonly reported issues [39].

Twin studies indicated that 40–60% of FEDs demonstrate heritability [40]. In parallel, brain scans of affected persons and genome-wide association studies (GWAS) pointed to the fact that AN is primarily observed in families with perfectionist, obsessive, and competitive traits [41,42].

A significant gender bias is apparent in the reported incidence, with 90% of the affected youngsters being female [43]. However, the incidence of AN among boys was also taken into account in the latest DSM edition [34], with the exclusion of amenorrhea from the typical diagnostic criteria, in order to include the male population towards a less gender-biased diagnosis.

### 1.3. AN as an NP Manifestation in SLE

Although AN was not included in the initial or the updated European Alliance of Associations for Rheumatology (EULAR) recommendations for the management of SLE with NP manifestations [27,44,45], research suggests that these two conditions often coincide. Cohorts of patients with FEDs show an increased risk of the development of autoimmune diseases, including SLE [46], supporting the hypothesis that a link appears to exist between immune-mediated mechanisms and the development of FEDs. According to Sirufo [47], AN and autoimmune diseases share a bidirectional relationship, since they both rely on common immunopathological pathways. Based on psycho-neuro-immuno-endocrinology [48], psychological stressors can orchestrate the mobilization of immune cells and promote the production of various inflammatory mediators [49,50], altering the immune response to viral or autoimmune challenges.

The present narrative review aimed to identify relevant original research and present cases of pediatric patients with AN and SLE, in an effort to understand possible links behind the two entities.

## 2. Methodology

### 2.1. Search Strategy

A detailed search was conducted in PubMed up to December 2022, for original research presenting the cases of children and adolescents with SLE and AN. Included keywords were (SLE), (anorexia[MeSH]), (Anorexia Nervosa[MeSH]), (eating disorders), (FED), (lupus), (systemic lupus erythematosus[MeSH]), (jSLE), (juvenile SLE), (juvenile lupus), and (pediatric lupus).

Inclusion criteria involved (i) original research, (ii) presenting pediatric patients (<18 years of age), (iii) with AN and SLE diagnoses, in either order of occurrence. Exclusion criteria included (i) original studies with adult samples, (ii) with anorexia defined as loss of appetite instead of a DSM-based diagnosis, and/or (iii) diagnoses of other autoimmune rheumatic diseases, or non-systemic forms of lupus. Three researchers independently assessed all retrieved items and reached a consensus regarding their inclusion/exclusion.

### 2.2. Data Extraction

Data was extracted from each retrieved item in a predefined excel form, involving the number and characteristics of participants, diagnostic criteria and occurrence of either diagnoses, prognosis and therapeutic approaches (whenever reported), and the detection of auto-antibodies.

## 3. Results

### 3.1. Case Reports and Case Series of Pediatric Patients with AN and SLE

A total of three case reports [51,52,53] and one case series [54] were retrieved from the scientific literature. Table 1 describes the available scientific literature presenting case reports of pediatric patients with SLE and AN.

Apart from the detailed primary research presented in Table 1, Sloan [55] also published the case of an adolescent with SLE who subsequently demonstrated AN, although the file could not be accessed. Furthermore, Nakamura and associates [56] also presented a case-report of an adolescent girl with jSLE and Sjögren’s syndrome, who had a psychiatric history of anorexia, although details regarding weight loss/gain were not provided.

In the presentation of a 13-year old girl with SLE and AN (Table 1), Bambery and associates [52] argued that a strategically placed hypothalamic lesion initiating the development of vasculitis in SLE might also propel the development of AN. However, the absence of more features pointing to dysfunction of the hypothalamus and the acute resolution of AN following the taper down of prednisolone (PRDL) did not corroborate this theory further [52].

Hyla-Klekot [51] (Table 1) presented a 16-year old girl exhibiting severe AN and a strict refusal to consume food, who subsequently exhibited symptoms of Raynaud’s phenomena and developed lupus, although the severity of AN manifestations induced a significant delay in the SLE diagnosis. Again, SLE activity and symptoms of AN were resolved following aggressive immunosuppressive therapy. In a similar case report [53], a 13-year-old girl with AN and depression as the preliminary diagnoses was found to have lupus cells in the bone marrow aspirate when a biopsy was performed to rule out leukemia due to peripheral cytopenia. SLE diagnosis was confirmed with high levels of antinuclear antibodies (ANA) and anti-double strand DNA (anti-dsDNA) [53]. Treatment with methylprednisolone relieved SLE symptoms and achieved weight gain.

Toulany and associates [54] presented a case series of seven children and adolescents, all attending the same Pediatric Rheumatology Clinic in Canada. One of them developed SLE with the diagnosis of AN following, while in the remaining six, AN diagnosis preceded that of SLE. Given that the total cases of AN among children and adolescents with an SLE diagnosis was 6 out of 425, the estimated prevalence of AN in jSLE was calculated as 1.4%. In all cases, SLE treatment resulted in improvement of AN symptomatology.

### 3.2. Coexistence of AN and Non-Systemic Forms of Lupus in Adolescents

In an early report, Hedrich [57] described the case of a 13-year-old boy with AN, manifesting typical Raynaud’s phenomena and a malar rash, who subsequently developed chilblain lupus erythematosus (CHLE).

Previous studies also reported pediatric patients with idiopathic chilblains and a body mass index (BMI) below the 5th percentile [58,59], as well as patients with Raynaud’s phenomena and AN [60]. The authors argued that the altered thermoregulation and the hyperreactive peripheral vascular response to the cold observed in AN—as a result of the lower resting metabolic rate—may drive perniosis and Raynaud’s phenomena in these patients [59,61].

Around that time, Pisani [62] presented a 13-year-old girl with AN and a subsequent diagnosis of discoid subacute lupus erythematosus (DLE). No data were provided on the prognosis of that girl.

Although CHLE and DLE do not represent systemic forms of lupus, they share common pathophysiological mechanisms and may eventually evolve into SLE, although such follow-up data were not provided either by Pisani [62] or Hedrich [57].

### 3.3. Timing of FEDs and SLE Onset

In SLE, impaired immunity takes place several years prior to the clinical onset of the disease, as manifested by the detection of autoantibodies [63]. Toulany [54] suggested that when considering the timing of AN’s clinical presentation in relation to the SLE diagnosis and the response of all patients to SLE therapy, it becomes apparent that AN may be another early manifestation of NPSLE. In fact, alterations in the concentrations of specific neurotransmitters and cytokines are important components of the AN pathophysiology [47]. On the other hand, patients with AN with joint symptoms, positive anti-nuclear antibodies, or lymphopenia should be further investigated and followed for the possible development of SLE [54]. Nonetheless, in eight out of the ten case reports presented in Table 1, AN preceded the diagnosis of lupus, although in all cases both diagnoses occurred within a time span of two years, suggesting the existence of common precipitating factors.

Furthermore, for jSLE, in particular, the timing of onset coincides with a period of increased concerns about body weight and body image among adolescents, enhanced by the use of social media and the “fragile” teen psychology. Thus, peri-adolescence is the period where both entities (jSLE and AN) peak, with both exhibiting increased inflammation markers and a variety of mental health symptoms. In this manner, it is not uncommon for either diagnosis to lead to the gradual development and/or presentation of the other.

## 4. Discussion

The present review presents several case reports and a case series of patients with concomitant jSLE and AN diagnoses, revealing that the two entities are often encountered together. In parallel, further reports were identified with adolescent patients who were diagnosed with AN and non-systemic forms of lupus.

### 4.1. Possible Links between SLE and AN

#### 4.1.1. Medication-Induced Changes in Body Weight and Body Image

Research has shown that children and adolescents with autoimmune or autoinflammatory diseases exhibit a greater risk (HR: 37%) of developing AN and other FEDs [64]. Zerwas et al. [64] were the first to demonstrate a bidirectional and multigenerational link between FEDs and diseases of innate and humoral immunity, using large population-based cohorts of children and adolescents [65]. In fact, according to a recent rapid review [66], autoimmune reactions consist of an established risk factor for the development of AN. Evidence suggests that in adolescents with SLE, steroid-induced changes in body weight and shape might act as triggers for the development of body dissatisfaction and AN [54]. According to Calzza [67], the use of corticosteroids (CS), disordered eating habits, and low physical activity levels may increase the accumulation of body fat in SLE. Indeed, in the available literature, PRDL-induced body weight accumulation was identified as a potential trigger for the development of AN [54,55,62]. Interestingly, in the Bambery [52] case report, the girl had been on PRDL treatment since the diagnosis of lupus was established, gradually developing central obesity and gaining weight. This alarmed her parents and siblings, who reproached her about being more cautious regarding weight gain and the amount of food consumed [52]. Unfortunately, this cascaded into an accelerated loss of 10 kg of body weight during the next 5 months. Similarly, in the Canadian case report [54], the girl was teased by classmates for gaining weight, which triggered body image dissatisfaction and fear of being fat, and heightened the desire to lose weight.

Research has revealed that a significant number of adolescents with jSLE become overweight/obese after CS treatment [68]. In parallel, it has been estimated that 25% of adolescents with lupus are obese, demonstrating poorer sleep quality, worse physical, social and emotional functioning, and higher serum tumor necrosis factor-α (TNF-α) levels, while experiencing more pain [69,70,71]. CS-induced obesity is highly dependent on gender, baseline BMI, and cumulative CS dosage [68]. In parallel, the growth delay as a result of CS [72] may induce further increments in the BMI of patients, complicating body image perception. Research on adults with SLE has revealed poor body image perception and a significant body image disturbance associated with anxiety, fatigue and depressive symptoms, low quality of life, and augmented pain sensation [73,74,75,76]. On the other hand, adolescent girls with jSLE have been suggested to have heightened appearance concerns compared to their healthy peers [77], representing a possible residue of medication-induced body weight accumulation. Since patients with SLE often exhibit nausea, anorexia, and vomiting, caused by the disease itself [78], it is often difficult to differentiate the exact symptoms of AN versus those of lupus.

Given that CS therapy is the mainstay of SLE treatment, and considering the observed body weight accumulation at the onset of lupus therapy, Manaboriboon [68] suggested that dietary advice and nutrition education should be provided to all patients with jSLE at diagnosis, with preventive obesity strategies, focusing particularly on adolescents with an elevated BMI at the time of disease onset. Since lupus is mainly presented in girls, who may be more sensitive to achieving and maintaining a “slim” appearance, adolescent patients must also be reassured that if a healthy diet is followed, obesity is not a definite outcome of CS treatment [68].

#### 4.1.2. Medication-Induced Psychiatric Effects

Aside from increased body weight gain, CS use is also associated with a variety of adverse psychiatric events, including anxiety, agitation, psychosis, insomnia, catatonia, depression, mood and cognitive changes, euphoria, depersonalization, delirium, dementia, and hypomania [79,80,81]. In this manner, according to Alpert [82], CS use can be both the cause and the treatment of NP symptoms in children with lupus, and thus the risk-benefit ratio of CS use must be carefully weighed for each patient. In any case, it is highly likely that these NP symptoms drive the development of AN in adolescents with juvenile lupus and that AN represents the epiphenomenon of a more aggressive CS therapy, in association with other corroborating environmental predisposing factors, including weight gain, body image concerns, etc.

#### 4.1.3. Psychological Stress Associated with Chronic Disease

Another theory points to the stress and psychological burden associated with a diagnosis of chronic illness during childhood or adolescence, which may impact psychological and physiological development [54,83,84]. According to research, accumulated chronic conditions are associated with anxiety and depression [85], both of which may precipitate FED, including AN manifestations [86]. Furthermore, research indicates that a variety of chronic diseases are associated with an increased risk of developing FEDs or disordered eating behaviors [87,88,89,90,91]. It is unclear, however, if this association is merely coincidental, or if specific medical conditions may predispose patients to the development/expression of specific FEDs [54].

In particular, in chronic conditions, where attaining a healthy diet is important for a better prognosis, such as in diabetes mellitus or lupus, patients appear particularly prone to the development of FEDs or other-specified FEDs (OSFEDs), including AN or orthorexia nervosa [86,87,88,92,93]. Thus, the goal of achieving health and controlling body weight may be the initial motive behind the adherence to a stricter diet, but this often takes the opposite turn, developing into a FED. Unfortunately, with the underlying NP symptoms, this result can be relatively common for children and adolescents with jSLE, with AN often manifesting in the years following SLE diagnosis.

#### 4.1.4. Chronic Inflammation in AN as a Trigger for SLE

In a recent study, among all patients attending a pediatric rheumatology clinic, 22.4% exhibited disordered eating patterns [94]. Of these, only 15.7% of the cohort had previously received a formal FED diagnosis. Complaints of abdominal pain, gastrointestinal comorbidities, and anxiety were among the most important predictors of disordered eating behaviors in the cohort [94]. Meta-analyses showed that patients with AN exhibited elevated levels of proinflammatory cytokines, including TNF-α, interleukin (IL)-1α, IL-10, epidermal growth factor (EGF), interferon (IFN)-γ, IL-6, and IL-1β in comparison to healthy controls, indicating the development of a chronic proinflammatory status [95,96,97], which in turn may have propelled the SLE manifestations. In fact, altered hormonal responses, as commonly observed in AN, have been shown to negatively influence the stress circuit through specific inflammation pathways [47].

#### 4.1.5. The Possible Role of Leptin and Shared Autoantibodies

Research indicates that children and adolescents with autoimmune or autoinflammatory diseases have a greater risk of developing AN [64], while, on the other hand, people with AN exhibit increased odds of being diagnosed with SLE [46,92]. Several possible pathways have been proposed to explain these phenomena. It is well known that patients with SLE have an increased risk for the development of mood disorders [98] and a high prevalence of NP manifestations [99]. In parallel, reduced absolute numbers and impaired functions of regulatory B cells (B_reg_) have been observed in most autoimmune diseases—including SLE [100,101]—, as well as in AN [102], pointing towards the existence of an impaired B_reg_ cell compartment function that heightens the risk of developing autoimmunity in AN. Furthermore, AN and SLE appear to be characterized by the presence of specific brain-reactive autoantibodies, which are responsible for the development of NP disorders among those with SLE, as well as being responsible for the development of SLE in patients with mental disorders [46]. Given the increased production of autoantibodies observed in patients with SLE, it is highly possible that some antibodies may also act against specific neurotransmitters, participating in food and appetite regulation [51].

Previous studies have corroborated this theory, by showing that core psychiatric and behavioral disorders in FEDs tend to correlate with levels of neuropeptide autoantibodies, including autoantibodies against α-melanocyte-stimulating hormone (α-MSH), pointing to the fact that AN is possibly related to autoantibody-mediated dysfunction of the melanocortin system [103,104,105,106]. Interestingly, research in mice revealed that treatment with α-MSH improved SLE disease activity [107], adding melanocortin peptides to the panel of potential therapeutic targets in SLE [108]. In parallel, gut dysbiosis mediates the production and regulation of such autoantibodies [109], suggesting that CS-induced dysbiosis [110,111] may well be a contributing factor to the development of AN traits.

Patients with SLE demonstrate increased serum concentrations of the satiety hormone leptin [112], whereas leptin is also implicated in the promotion of SLE, by increasing the production of autoantibodies, exhibiting a strong correlation to disease activity and inhibiting immune regulation [112,113,114]. Furthermore, the incidence of SLE is greater among women than men, with the former exhibiting higher concentrations of leptin in general [114]. In parallel, IgG antibodies have been shown to interact with circulating ghrelin and leptin levels [115], while patients with AN often present autoantibodies directed to ghrelin and leptin.

#### 4.1.6. Shared Genetic Traits

According to research, a positive family history of autoimmune or autoinflammatory disorders in a first-degree relative is also associated with an increased risk of the development of FEDs in youth [64]. Recently, genome-wide association studies (GWAS) highlighted the importance of immune-mediated pathways in the development of EDs. The neural cell adhesion molecule-2 (*NCAM2*) gene, which was identified in an analysis of patients with AN, encodes proteins belonging to the immunoglobulin superfamily [116]. Furthermore, a close link has been shown to exist between AN and the immune system [117], with the identification of a significant locus for AN situated on chromosome 12, SNP rs4622308, which was previously shown to be a locus for autoimmune disorders (including rheumatoid arthritis) [102,118].

#### 4.1.7. Adverse Childhood Experiences (ACEs) as Triggers of AN and SLE

Adverse childhood experiences (ACEs) include traumatic and life-stressor events of a heterogenous nature, associated with lower psychological resilience at a young age [119], health risk behaviors and poor health in later life [120,121,122,123,124], immune dysregulation and immune system inflammation [124,125,126], as well as with a negative impact on the long-term developmental trajectory [123,127]. Inevitably, the diagnoses of both SLE and AN has also been researched in the context of ACEs. Experiencing ACEs has been shown to disrupt immunity [128,129], increasing the risk of the onset of autoimmune diseases, and rheumatic diseases in particular [129,130,131,132]. In SLE, a history of ACEs has been associated with poorer disease outcomes, greater NP involvement, and worse perceived health status [133,134]. On the other hand, as trauma also impacts mental health [135,136], patients with FEDs report experiencing more ACEs compared to the rest of the population, with severer forms of trauma being related to aggravated FED symptomatology [137,138,139,140,141,142]. Taken together, it appears that ACEs may trigger the development of both entities, indicating another possible route for a dual AN–jSLE diagnosis.

### 4.2. Strengths and Limitations of the Present Work

Due to the nature of the present review (narrative), selection bias might exist in the presented work, although efforts were made to limit this source of bias through the performance of a systematic and thorough search. On the other hand, non-systematic reviews are also important components of the scientific literature, as they can aid both experts and non-experts in triaging and understanding the increasing volume of original research that is being published in scientific journals [143,144,145]. In fact, according to Bastian [146], narrative reviews form the basis of medical literature synthesis.

Although, at the moment, the scientific literature only presents individual case reports and a case-series of adolescents with jSLE and AN, the coexistence of the two conditions does not appear to be rare. Several additional cases have been reported among adult patients, some with non-systemic forms of lupus [147], while many more have also been published in the non-scientific press [148,149,150]. Furthermore, the rarity of both conditions makes the conduction of relevant studies more difficult.

### 4.3. Clinical Implications of the Findings

According to Toulany [54], the calculated prevalence of AN in adolescents with jSLE (1.4%) greatly exceeds that of their SLE-free peers, indicating that AN most likely consists of a NP manifestation of lupus, rather than the expected incidence in this particular age group. Thus, it appears that a shared underlying mechanism or an additional mediating variable may influence the risk of developing and expressing both diseases [92]. It is also possible that each disease entity mimics the symptoms of the other, complicating the diagnosis.

From the health professional’s point of view, educating rheumatologists on the signs and symptoms of AN is an important step for the early identification of cases and the prompt initiation of evidence-based therapeutic approaches. The management of really sick patients with anorexia nervosa (MARSIPAN) report and the junior MARSIPAN guidelines [151,152,153] can aid in the identification of early warning signs of AN. Nutritional education performed by experienced dietitians at jSLE diagnosis may also reduce the weight accumulation associated with CS use and help patients improve growth and body image. In parallel, it would also be prudent for clinicians to include questions targeted to SLE when assessing young patients with AN. If any symptoms or physical signs that raise suspicion of SLE are identified, they should request appropriate laboratory investigations and potentially seek a rheumatology opinion from experts. Inversely, for patients with jSLE, there should be low threshold for suspecting AN, especially in the context of other NP manifestations.

Nonetheless, more research is required, ideally at a cohort level, to understand the extent of the problem and take the necessary measures to provide recommendations. It suffices to say, the EULAR and the American College of Rheumatology (ACR) should consider the inclusion of AN, and if required, additional FEDs in the list of NP manifestations associated with SLE.

## 5. Conclusions

Previous research has suggested the existence of a close link between FEDs and autoimmunity, and herein we provide further evidence to support this relationship, by presenting and discussing all published case reports and case series of adolescent patients with coexisting AN and jSLE. Future research should aim at prospectively assessing the prevalence of AN in jSLE, from the time of initial diagnosis, in order to clarify this relationship further and help us understand the mechanistic drivers and prerequisites of this association.

## Figures and Tables

**Table 1:** Case reports/series published in the literature describing pediatric patients with SLE and AN.

First Author	Origin	Study Design	Participants	Order of Diagnoses	AN Diagnosis	Autoantibodies	Results
Hyla-Klekot [51]	Poland	Case study	16-year-old girl severe AN, followed by immunological and clinical manifestations of RP and jSLE	AN preceded SLE diagnosis	DSM-V	ANA+, anti-dsDNA and anti-nuc	The severity of AN symptoms delayed the diagnosis of SLE, which was already active. Immunosuppressive therapy decreased lupus activity and resolved the symptoms of AN.
Bambery [52]	India	Case study	13-year-old girl with SLE who developed AN 1-year post-diagnosis	SLE preceded AN diagnosis	DSM-III	NR (SLE cells and ANF were assessed instead)	A comprehensive treatment program with family therapy, psychotherapy, medical, dietary (1800 kcal/day high-protein diet), physical and behavioral therapy, as well as contingency contracting was initiated and PRDL was gradually tapered down. Improvement was sustained and menstruation was resumed.
Xu [53]	USA	Case study	A 13-year-old girl with AN and depression as the preliminary diagnoses	AN preceded SLE diagnosis		ANA > 12 U and anti-dsDNA > 1000 IU/mL	Due to peripheral cytopenia, a bone marrow biopsy was performed and LE cells were found in the bone marrow aspirate. Further testing demonstrated high levels of ANA (>12 U) and anti–dsDNA (>1000 IU/mL), confirming SLE diagnosis. Treatment with mPRED quickly improved the symptoms. A month post-discharge she had gained 6.1 kg.
Toulany [54]	Canada	Case series	Seven children/adolescents with jSLE and AN (restrictive subtype) out of 425 in total attending the pediatric rheumatology clinic	AN preceded SLE diagnosis for *n* = 6 and SLE preceded AN diagnosis for *n* = 1.	DSM-IV	All were ANA+, 2 had anti-dsDNA, two had anti-Ro, one also had anti-La, ACA and RF, and one had anti-Sm and anti-RNP.	One patient developed AN at 15 months post-SLE diagnosis, attributed to PRDL-induced BW gain and a cushingoid body appearance. Among the remaining 6 patients, the age of AN symptoms’ onset was 12.2 years and the diagnosis of AN appeared at 13.6 years. The age at SLE diagnosis was 14.2 years, at 20 months post-onset of AN symptomatology. SLE treatment improved AN symptoms in all patients.

ACA—anti-cardiolipin; AN—anorexia nervosa; ANA—antinuclear antibodies; ANF—antinuclear factor; anti-La—antibodies to the La antigen; anti-nuc—anti-nucleosome antibodies; anti-RNP—anti-ribonuclear protein; anti-Sm—anti-Smith; dsDNA—double strand DNA; Ca—calcium; CS—corticosteroids; DSM—diagnostic and statistical manual for mental disorders; jSLE—juvenile SLE; NR—not reported; LE—lupus erythematosus; mPRED—methylprednisolone; PRDL—prednisolone; RF—rheumatoid factor; RP—Raynaud’s phenomenon; SLE—systemic lupus erythematosus.

## Data Availability

All available data are presented within the manuscript text.
